# Case report: A primary calcified cardiac mass in right atrium partially obstructs the tricuspid valve in a patient on hemodialysis

**DOI:** 10.3389/fcvm.2022.950628

**Published:** 2022-08-16

**Authors:** Hongduan Liu, Xiaokang Tu, Hao Zhang, Chengming Fan, Haoyu Tan, Long Song, Qin Wu, Liming Liu

**Affiliations:** Department of Cardiovascular Surgery, The Second Xiangya Hospital, Central South University, Changsha, China

**Keywords:** cardiac surgery, chronic kidney disease, calcified cardiac tumor, tricuspid valve, hemodialysis

## Abstract

Primary cardiac calcification is a rare benign mass in patients with end-stage renal disease. A few cases have been reported in the literatures. In this case study, during a routine checkup for hemodialysis, a transthoracic echocardiography on a 19-year-old male showed a cardiac mass in the right atrium that was partially obstructing the tricuspid valve. Cardiac magnetic resonance imaging showed a well-circumscribed, homogeneous “shadow” in the right atrium; it measured 29 × 27 mm, had equal T1- and T2-weighted signal intensities, and was adjacent to the tricuspid valve. According to 18F-fluorodeoxyglucose positron emission tomography combined with computed tomography, there was a dense circular shadow in the right atrium abutting the tricuspid valve, but there was no increase in glucose metabolism. Median sternotomy was performed for the surgical resection of the mass, and a cardiopulmonary bypass was completed. The mass was completely removed. The patient recovered well and was discharged 10 days after the surgery. Histological examination showed that the mass contained multiple calcified nodules. No mass recurrence was found by echocardiography during the 12th-month follow-up.

## Introduction

Most primary cardiac tumors are myxomas, lipomas, and fibroelastomas, of which calcification is found in cardiac myxoma, thrombosis, and osteosarcoma, respectively (1, 2). A cardiac calcified amorphous tumor (CCAT), a rare non-neoplastic intracavitary cardiac tumor, is characterized by calcification deposits in an amorphous background with fibrin materials and focal inflammation (3). Solely calcified cardiac masses without inflammation are extremely rare non-neoplastic cardiac masses that mimic malignancy on imaging. In particular, calcified cardiac masses are highly unusual in patients with end-stage renal disease (ESRD) who are on hemodialysis. Herein, a rare case of an atypical cardiac calcified mass partially obstructing the tricuspid valve in a patient on hemodialysis is presented.

## Case presentation

A 19-year-old male was referred to our hospital because a cardiac mass in the right atrium was detected by transthoracic echocardiography (TEE). The patient presented with cough and resting dyspnea and had a history of hypertension and ESRD, requiring hemodialysis for 2 years and hyperparathyroidism for 1 year. Two years ago, the urine culture was positive for C.parapsilosis, and then the fluconazole had been administrated until the urine culture was negative. Nephrotic syndrome and uremia of chronic kidney failure (CKD) were diagnosed by a kidney biopsy due to nocturia and elevated creatinine. Hemodialysis was being performed three times a week. An arteriovenous fistula was performed without central venous catheterization in the left upper extremity 1 year ago to improve the routine hemodialysis. Physical examination showed a normal temperature, 123/78 mmHg blood pressure, and 105 bpm heart rate. There was no cardiac murmur or family history of cardiovascular disease. Mild edema was found in the lower limb. Creatine was 1,015 μmol/L, N-terminal pro-B-type natriuretic peptide was 1,796 pg/ml, hemoglobin was 112 g/L, and parathyroid hormone level was above 2,000 pmmol/L. Laboratory data revealed a calcium level of 2.37 mmol/L and phosphorus level of 2.36 mmol/L. The other laboratory test results were normal.

**Figure 1:**
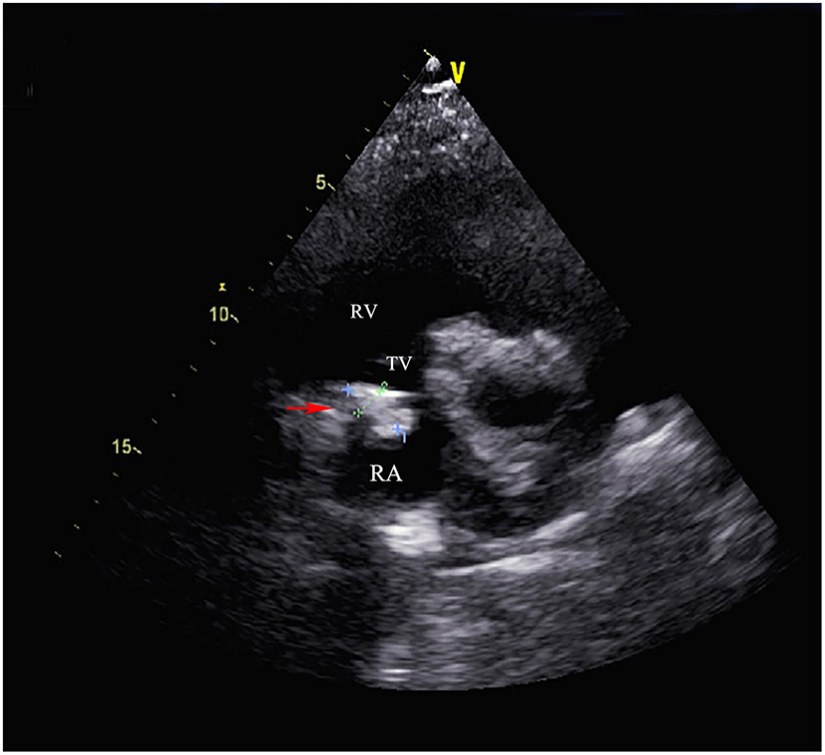
Transthoracic echocardiography showed the cardiac mass (30 mm x 28 mm) located in the right atrium and the anterior tricuspid valve was partially obstructed by the mass but the flow velocity of tricuspid valve did not accelerate. (RA, right atrium; RV, right ventricle; TV, tricuspid valve).

**Figure 2:**
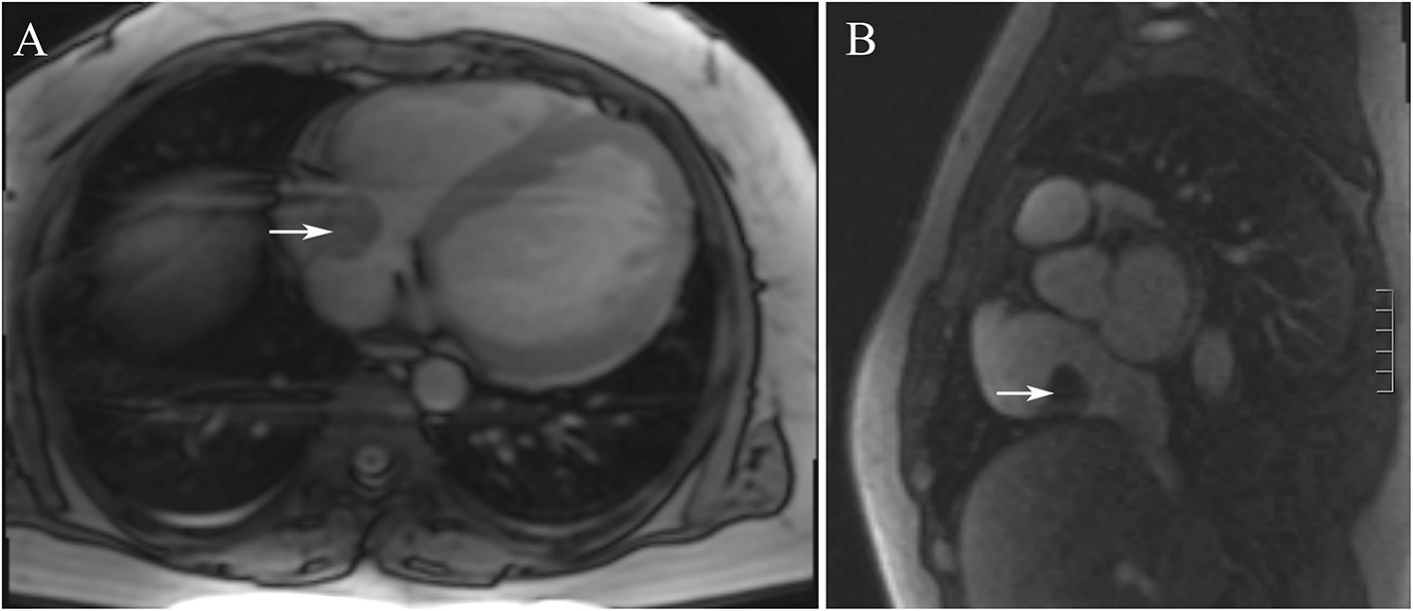
**(A,B)** The cardiac magnetic resonance imaging showed that showed that a well-circumscribed, homogenous “shadow” in the right atrium; it measured 29 mm × 27 mm, had equal T1- and T2-weighted signal intensities, was adjacent to the tricuspid valve.

**Figure 3:**
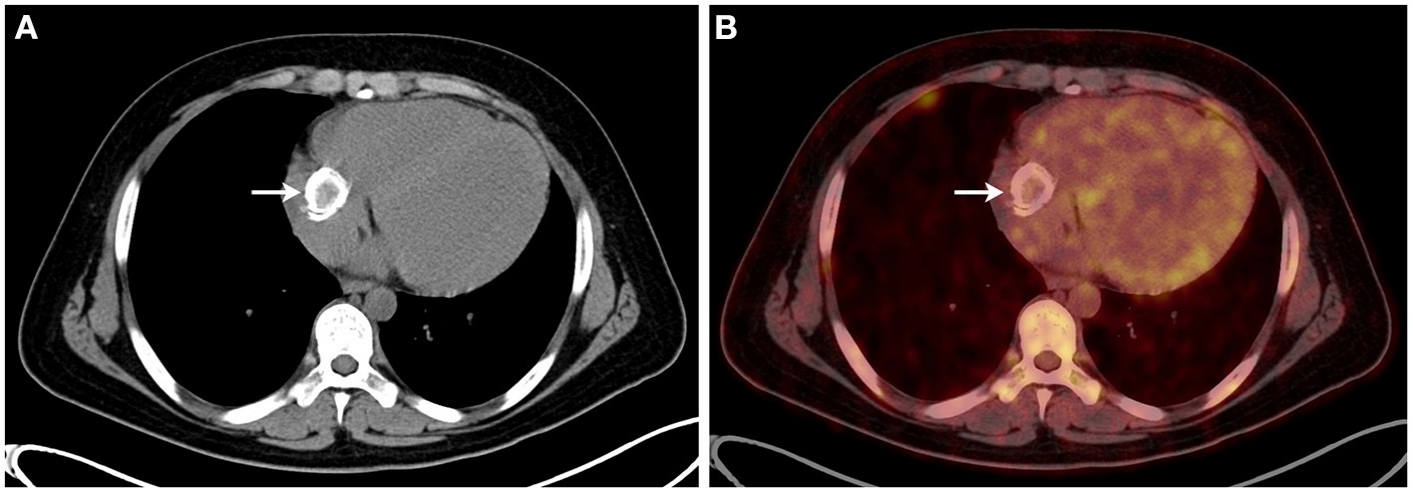
**(A,B)** The 18F-fluorodeoxyglucose positron emission tomography combined with computed tomography showed that a dense circular shadow was in the right atrium abutting the tricuspid valve (32 × 34 mm), and there was no increase in glucose metabolism.

**Figure 4:**
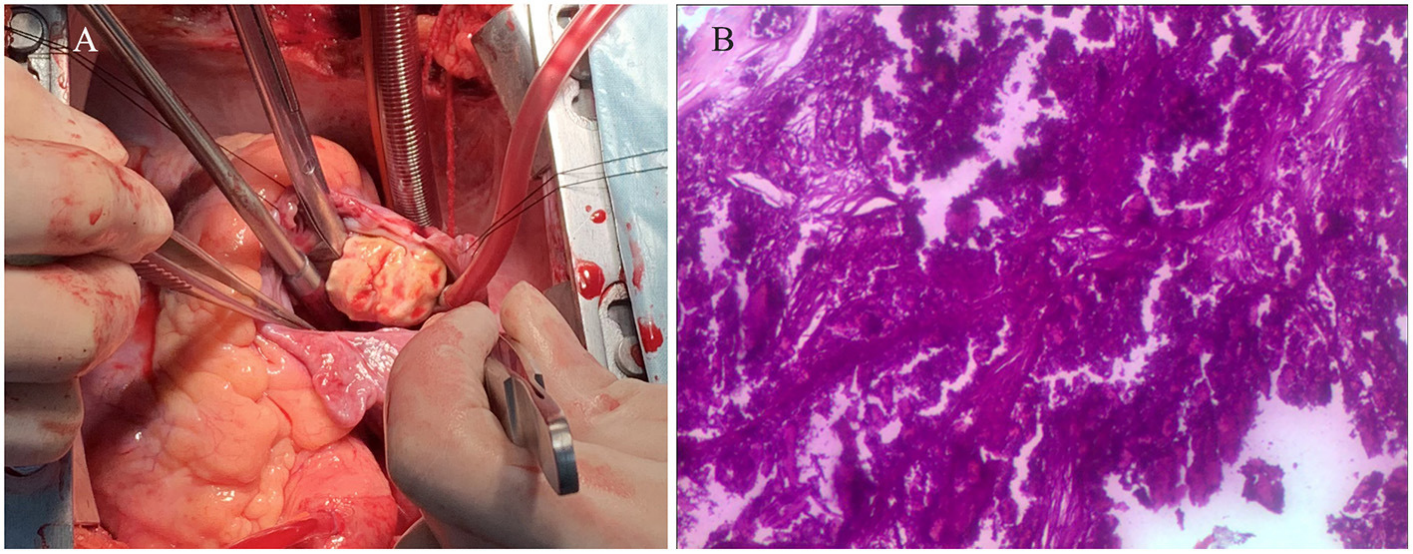
**(A)** The intraoperative image showed that the mass, with a firm-hard texture, was confirmed to be partially embedded in the right atrium. **(B)** The histopathological examination revealed that the mass consisted of abundant calcific deposits without malignant cells.

Electrocardiography showed sinus tachycardia. TTE detected the cardiac mass (30 x 28 mm) in right atrium. The LVEF was 39% and FS was 19. The anterior tricuspid valve was partially obstructed by the mass, but the flow velocity of tricuspid valve did not accelerate (Figure 1). Cardiac magnetic resonance imaging (cMRI) showed that a well-circumscribed, homogenous “shadow” in the right atrium; it measured 29 mm × 27 mm, had equal T1- and T2-weighted signal intensities, was adjacent to the tricuspid valve (Figure 2). According to 18F-fluorodeoxyglucose (FDG) positron emission tomography combined with computed tomography (PET–CT), a dense circular shadow was in the right atrium abutting the tricuspid valve (32 × 34 mm). There was no increase in glucose metabolism, and the tumor was considered benign (Figure 3). There was no calcium deposited in the coronary arteries or other organs. The initial clinical diagnosis was a right atrial myxoma or fibroelastoma. Median sternotomy was performed to remove the mass and eliminate the obstruction of the tricuspid valve. A cardiopulmonary bypass was conducted using ascending aortic and superior and inferior vena cava cannulation. Without cardiac arrest, the right atrium was opened, and the mass, with a firm-hard texture, was confirmed to be partially embedded in the right atrium (Figure 4A). It was removed together with the implantation base. Histopathological examination revealed that the mass consisted of abundant calcific deposits without malignant cells (Figure 4B). The patient's postoperative course was uneventful, involving only three-times-a-week hemodialysis. The postoperative echocardiography showed that the LVEF was 55% and there was no residual of the mass. The patient was found to have recovered well and without recurrence at the 12th-month follow-up.

## Discussion and conclusion

Primary cardiac tumors with calcification occur in cardiac myxoma, thrombosis, and osteosarcoma, among others. A CCAT is characterized by a calcified nodule in an amorphous background with fibrous degeneration and focal inflammation (3). Solely calcified cardiac mass without thrombus or inflammation are extremely rare (4). A past study showed that inflammation does not occur in CCATs in non-ESRD (5). This is consistent with our findings: the mass consisted only of abundant calcific deposits. The novelty of our case is that the mass presented in a patient with ESRD who was on hemodialysis.

The etiology of the CCATs is unknown (6), but some cases are associated with the hemodialysis in ESED (7–11). Metabolic disorders of calcium and phosphorus, which are common comorbidities among patients with CKD requiring hemodialysis, may result in calcification deposits in the mass, vascular calcification, and calciphylaxis (12–14). Hyperparathyroidism is the main comorbidity of CKD, especially among patients requiring hemodialysis (15). As a result, metabolic disorders of calcium and phosphorus may result in calcium deposition in the vascular system via the disruption and alteration of the calcium regulatory mechanisms (16). Our study presents an extremely rare case: a calcified right atrial mass in a patient with ESED and hyperparathyroidism who was on hemodialysis.

Categorization of the symptoms depends on their location, obstruction, or embolization. Most patients are asymptomatic, and symptoms include shortness of breath, syncope (17), stroke (18), and central retinal arterial occlusion (6). In our case, cough, resting dyspnea, and lower limb edema were presented, which may have been related to low partially obstruction of the tricuspid valve and uremia. Calcified primary cardiac tumors have been found in all four chambers, usually in the mitral valve and annulus (7, 19, 20). CCATs presented most frequently in the right atrium and ventricle in non-ERSD patients, whereas CCTAs in the mitral annulus are significantly more common in patients with ESRD than in patients without ESRD (19). By contrast, the present study shows an exceptional case where a calcified cardiac mass is presented in the right atrium abutting the tricuspid valve in a ESRD on hemodialysis.

Appropriate screening imaging modalities for cardiac mass include echocardiography, computed tomography (CT), cMRI, and PET-CT; they can be used to make differential diagnoses among myxomas, fibroelastomas, thrombi, and among others. All of these can eliminate the problem of differential diagnoses (4). Although PET-CT and FDG uptake can provide valuable information about inflammation for the differential diagnoses (5), histopathology is still needed to make a final diagnosis, especially for CCATs. In the current study, a clinial diagnosis of a benign tumor was made, based on the cMRI, echocardiographic, and PET-CT findings. If a patient presents with symptoms caused by a mass or in cases where the histopathology is uncertain in relation to the tumor, surgical resection should be performed to alleviate the symptoms, reduce the risks, and clarify the nature of the mass.

In conclusion, CCAT is a rare non-neoplastic primary cardiac tumor characterized by calcification deposits with fibrin materials and inflammation. Solely calcified cardiac masses without inflammation are an exceptional subtype of CCATs. Abnormal calcium metabolism due to renal dysfunction and the inflammation associated with hemodialysis may contribute to the occurrence of the CCATs. Conventional imaging modalities, such as TTE, CT, and cMRI cannot specifically define the CCATs. Surgical resection is an appropriate measure for relieving symptoms, reducing the risks, and clarifying the pathological diagnoses of patients with CCATs.

## Data availability statement

The original contributions presented in the study are included in the article/supplementary material, further inquiries can be directed to the corresponding author.

## Ethics statement

The studies involving human participants were reviewed and approved by Ethics Committee of the Second Xiangya Hospital. The patients/participants provided their written informed consent to participate in this case study. Written informed consent was obtained from the individual(s) for the publication of any potentially identifiable images or data included in this article.

## Author contributions

HL drafted the manuscript. HL and XT designed the study. HL, HZ, and LL performed the surgery. LS, CF, HT, and LL revised the manuscript. HL, XT, and QW were responsible for the collection of data or analysis. All authors read and approved the final manuscript.

## Conflict of interest

The authors declare that the research was conducted in the absence of any commercial or financial relationships that could be construed as a potential conflict of interest.

## Publisher's note

All claims expressed in this article are solely those of the authors and do not necessarily represent those of their affiliated organizations, or those of the publisher, the editors and the reviewers. Any product that may be evaluated in this article, or claim that may be made by its manufacturer, is not guaranteed or endorsed by the publisher.
